# Bis{2-[(2-hy­droxy-2-methyl­prop­yl)imino­meth­yl]-4-nitro­phenolato}nickel(II) dimethyl­formamide monosolvate

**DOI:** 10.1107/S1600536811031229

**Published:** 2011-08-06

**Authors:** Kouassi Ayikoé, Yilma Gultneh, Ray J. Butcher

**Affiliations:** a Department of Chemistry, Howard University, 525 College Street NW, Washington, DC 20059, USA

## Abstract

In the title compound, [Ni(C_11_H_13_N_2_O_4_)_2_]·C_3_H_7_NO, the Ni^II^ ion is octa­hedrally coordinated in an N_2_O_4_ environment by two identical Schiff base ligands. The Ni—O bond lengths range from 2.004 (2) to 2.106 (2) Å, while the Ni—N bond lengths are 2.038 (2) and 2.0465 (19) Å. The *cis* bond angles range from 78.64 (8) to 97.30 (8)°, with the former being due to the small bite of the amino­alcohol ligand, while the *trans* bond angles range from 167.86 (8) to 171.23 (8)°. One of the alcohol H atoms forms a hydrogen bond with the dimethyl­formamide (DMF) solvent mol­ecule, while the other links mol­ecules into chains along the *b* axis through inter­molecular O—H⋯O hydrogen bonds. There are bifurcated C—H⋯O inter­actions involving one of the nitro groups between parallel stacks of mol­ecules in the *b*-axis direction.

## Related literature

For similar nickel Schiff base complexes, see: Ali *et al.* (2006 ▶); Butcher *et al.* (1981 ▶, 2009 ▶); Gultneh *et al.* (1998 ▶); Mustafaa *et al.* (2009 ▶); Zhang *et al.* (2010 ▶).
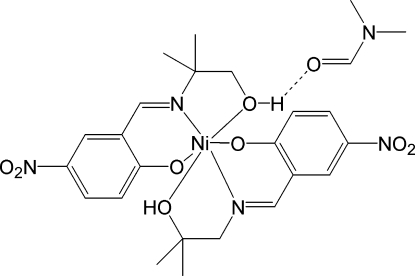


## Experimental

### 

#### Crystal data

[Ni(C_11_H_13_N_2_O_4_)_2_]·C_3_H_7_NO
*M* *_r_* = 606.27
Monoclinic, 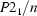

*a* = 11.42279 (16) Å
*b* = 11.42936 (18) Å
*c* = 21.4903 (3) Å
β = 99.1120 (14)°
*V* = 2770.26 (7) Å^3^
*Z* = 4
Cu *K*α radiation
μ = 1.54 mm^−1^
*T* = 295 K
0.44 × 0.21 × 0.18 mm

#### Data collection

Oxford Diffraction Xcalibur Ruby Gemini diffractometer
Absorption correction: multi-scan (*CrysAlis PRO*; Oxford Diffraction, 2009 ▶) *T*_min_ = 0.650, *T*_max_ = 1.000
13929 measured reflections
5826 independent reflections
4514 reflections with *I* > 2σ(*I*)
*R*_int_ = 0.026

#### Refinement

*R*[*F* ^2^ > 2σ(*F* ^2^)] = 0.055
*wR*(*F* ^2^) = 0.174
*S* = 1.05
5826 reflections
367 parameters
H-atom parameters constrained
Δρ_max_ = 0.56 e Å^−3^
Δρ_min_ = −0.53 e Å^−3^

### 

Data collection: *CrysAlis PRO* (Oxford Diffraction, 2009 ▶); cell refinement: *CrysAlis PRO*; data reduction: *CrysAlis PRO*; program(s) used to solve structure: *SHELXS97* (Sheldrick, 2008 ▶); program(s) used to refine structure: *SHELXL97* (Sheldrick, 2008 ▶); molecular graphics: *SHELXTL* (Sheldrick, 2008 ▶); software used to prepare material for publication: *SHELXTL*.

## Supplementary Material

Crystal structure: contains datablock(s) I, global. DOI: 10.1107/S1600536811031229/jj2096sup1.cif

Structure factors: contains datablock(s) I. DOI: 10.1107/S1600536811031229/jj2096Isup2.hkl

Additional supplementary materials:  crystallographic information; 3D view; checkCIF report

## Figures and Tables

**Table 1:** Hydrogen-bond geometry (Å, °)

*D*—H⋯*A*	*D*—H	H⋯*A*	*D*⋯*A*	*D*—H⋯*A*
O2*A*—H2*A*⋯O1*S*	0.82	1.94	2.734 (5)	163
O2*B*—H2*B*⋯O1*B*^i^	0.82	1.81	2.619 (3)	166
C9*A*—H9*AA*⋯O1*A*^i^	0.96	2.59	3.422 (4)	146
C11*A*—H11*B*⋯O3*B*^ii^	0.97	2.50	3.394 (4)	153
C5*B*—H5*BA*⋯O4*A*^iii^	0.93	2.57	3.418 (4)	151

Symmetry codes: (i) 
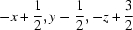
; (ii) 
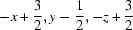
; (iii) 
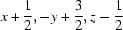
.

**Fig. 1.**
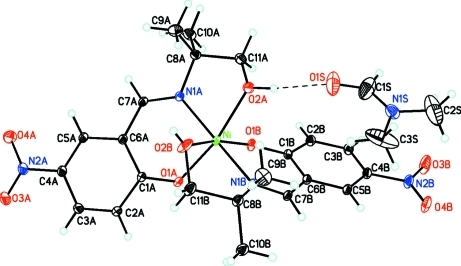
Diagram of C25H33N5NiO9, showing atom labeling. Atomic displacement parameters are at the 30% level. Hydrogen bonds are shown by dashed lines.

**Fig. 2.**
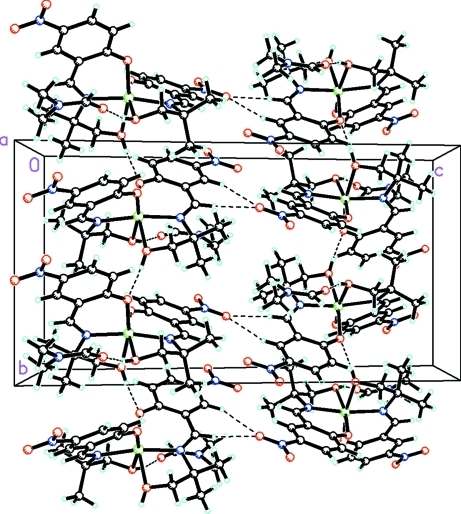
The molecular packing for C25H33N5NiO9 viewed down the a axis. Hydrogen bonds are shown by dashed lines.

## supplementary crystallographic information

### Comment

In the title complex two uninegative tridentate ligands, 5-nitro salicylaldehydene-2,2-dimethyl ethylimine-1-ol, coordinate to the nickel atom producing N_2_O_4_ in a slightly distorted octahedral coordination environment. The distortion can be justified by the bond angles formed by the coordinating atoms to the metal: N(1 A)—Ni—O(2 A) = 168.90 (8)°, N(1 A)—Ni—O(1B) = 98.60 (8)°, N(1 A)—Ni—O(2B) = 93.54 (8)°, N(1 A)—Ni—N(1B) = 171.23 (8)°, O(1B)—Ni—O(2B) = 167.86° and O(1 A)—Ni—O(2 A) = 168.90 (8)°. The Ni—O bond lengths are slightly shorter than the Ni—N bond lengths [Ni—N(1 A) = 2.038 (2) Å, Ni—N(1B) = 2.0465 (19) Å, Ni—O(2B) = 2.101 (2) Å, Ni—O(2 A) = 2.106 (2) Å] as is usually found in such complexes (Ali *et al.* 2006; Butcher *et al.* 1981, 2009; Gultneh *et al.* 1998; Mustafaa *et al.* 2009; Zhang *et al.* 2010).

One of the alcohol H atoms forms a hydrogen bond with the dimethylformamide solvate molecule while the other links the molecules into chains along the *b* axis through intermolecular O—H···O hydrogen bonds. There are bifurcated C—H···O interactions between parallel stacks of molecules in the *b* direction involving one of the nitro groups.

### Experimental

The complex, C_25_H_33_N_5_NiO_9_, was synthesized by adding a 20 mL solution of nickel nitrate hexahydrate (0.61 gram, 2.63 mmol) to another 20 ml of methanol solution containing 1.25 gram (5.26 mmol) of the ligand, 5-nitrosalicylaldehydene-2,2-dimethyl ethylimine-1-ol. Three drops of triethylamine was added followed by continuous stirring overnight at 40°C. The solution was then filtered, evaporated under vacuum, and washed with ethanol to yield 95% of a dark greenish yellow solid. About 0.20 mg of the sample was dissolved in DMF/MeOH (1:3) then layered with diethyl ether to give dark-yellow–green crystals.

### Refinement

H atoms were placed in geometrically idealized positions and constrained to ride on their parent atoms with a C—H distances of 0.93 to 0.97 Å and O—H distances of 0.82. Isotropic thermal parameters were *U*_iso_(H) = 1.2*U*_eq_(C) [*U*_iso_(H) = 1.5*U*_eq_(C)].

### Crystal data

**Table:**

[Ni(C_11_H_13_N_2_O_4_)_2_]·C_3_H_7_NO	*F*(000) = 1272
*M**_r_* = 606.27	*D*_x_ = 1.454 Mg m^−^^3^
Monoclinic, *P*2_1_/*n*	Cu *K*α radiation, λ = 1.54184 Å
Hall symbol: -P 2yn	Cell parameters from 6836 reflections
*a* = 11.42279 (16) Å	θ = 4.4–77.3°
*b* = 11.42936 (18) Å	µ = 1.54 mm^−^^1^
*c* = 21.4903 (3) Å	*T* = 295 K
β = 99.1120 (14)°	Needle, pale-green
*V* = 2770.26 (7) Å^3^	0.44 × 0.21 × 0.18 mm
*Z* = 4	

### Data collection

**Table:**

Oxford Diffraction Xcalibur Ruby Gemini diffractometer	5826 independent reflections
Radiation source: Enhance (Cu) X-ray Source	4514 reflections with *I* > 2σ(*I*)
graphite	*R*_int_ = 0.026
Detector resolution: 10.5081 pixels mm^-1^	θ_max_ = 77.5°, θ_min_ = 4.4°
ω scans	*h* = −13→14
Absorption correction: multi-scan (*CrysAlis PRO*; Oxford Diffraction, 2009)	*k* = −14→13
*T*_min_ = 0.650, *T*_max_ = 1.000	*l* = −26→17
13929 measured reflections	

### Refinement

**Table:**

Refinement on *F*^2^	Primary atom site location: structure-invariant direct methods
Least-squares matrix: full	Secondary atom site location: difference Fourier map
*R*[*F*^2^ > 2σ(*F*^2^)] = 0.055	Hydrogen site location: inferred from neighbouring sites
*wR*(*F*^2^) = 0.174	H-atom parameters constrained
*S* = 1.05	*w* = 1/[σ^2^(*F*_o_^2^) + (0.116*P*)^2^ + 0.4736*P*] where *P* = (*F*_o_^2^ + 2*F*_c_^2^)/3
5826 reflections	(Δ/σ)_max_ = 0.001
367 parameters	Δρ_max_ = 0.56 e Å^−^^3^
0 restraints	Δρ_min_ = −0.53 e Å^−^^3^

### Special details

**Table:**

Geometry. All e.s.d.'s (except the e.s.d. in the dihedral angle between two l.s. planes) are estimated using the full covariance matrix. The cell e.s.d.'s are taken into account individually in the estimation of e.s.d.'s in distances, angles and torsion angles; correlations between e.s.d.'s in cell parameters are only used when they are defined by crystal symmetry. An approximate (isotropic) treatment of cell e.s.d.'s is used for estimating e.s.d.'s involving l.s. planes.
Refinement. Refinement of *F*^2^ against ALL reflections. The weighted *R*-factor *wR* and goodness of fit *S* are based on *F*^2^, conventional *R*-factors *R* are based on *F*, with *F* set to zero for negative *F*^2^. The threshold expression of *F*^2^ > σ(*F*^2^) is used only for calculating *R*-factors(gt) *etc*. and is not relevant to the choice of reflections for refinement. *R*-factors based on *F*^2^ are statistically about twice as large as those based on *F*, and *R*- factors based on ALL data will be even larger.

### Fractional atomic coordinates and isotropic or equivalent isotropic displacement parameters (Å^2^)

**Table:**

	*x*	*y*	*z*	*U*_iso_*/*U*_eq_	
Ni	0.28214 (3)	0.69956 (4)	0.739938 (17)	0.04163 (16)	
O1A	0.13279 (18)	0.7923 (2)	0.73843 (9)	0.0627 (6)	
O2A	0.43631 (19)	0.5964 (2)	0.75995 (9)	0.0672 (6)	
H2A	0.4851	0.5947	0.7358	0.081*	
O3A	−0.2832 (2)	0.8371 (3)	0.88948 (13)	0.0905 (8)	
O4A	−0.1546 (3)	0.7581 (5)	0.95965 (12)	0.1320 (15)	
O1B	0.38319 (17)	0.84650 (17)	0.74068 (8)	0.0505 (4)	
O2B	0.1755 (2)	0.5507 (2)	0.71838 (10)	0.0850 (8)	
H2B	0.1676	0.4831	0.7297	0.102*	
O3B	0.7678 (3)	1.0122 (4)	0.58611 (14)	0.1350 (16)	
O4B	0.6486 (2)	0.9308 (3)	0.51235 (11)	0.0932 (9)	
O1S	0.6253 (5)	0.6221 (4)	0.6974 (3)	0.190 (3)	
N1A	0.29366 (18)	0.67706 (18)	0.83479 (9)	0.0418 (4)	
N2A	−0.1859 (2)	0.7931 (3)	0.90622 (13)	0.0739 (8)	
N1B	0.26685 (17)	0.69504 (17)	0.64379 (9)	0.0395 (4)	
N2B	0.6746 (2)	0.9632 (3)	0.56688 (13)	0.0737 (8)	
N1S	0.6828 (3)	0.6268 (4)	0.60657 (18)	0.0901 (10)	
C1A	0.0612 (2)	0.7858 (2)	0.77807 (11)	0.0433 (5)	
C2A	−0.0573 (2)	0.8288 (3)	0.76076 (13)	0.0530 (6)	
H2AA	−0.0803	0.8582	0.7203	0.064*	
C3A	−0.1370 (2)	0.8284 (3)	0.80106 (13)	0.0536 (6)	
H3AA	−0.2134	0.8566	0.7883	0.064*	
C4A	−0.1031 (2)	0.7851 (3)	0.86214 (13)	0.0524 (6)	
C5A	0.0081 (2)	0.7403 (3)	0.88111 (11)	0.0485 (6)	
H5AA	0.0282	0.7108	0.9217	0.058*	
C6A	0.0916 (2)	0.7383 (2)	0.84022 (11)	0.0421 (5)	
C7A	0.2064 (2)	0.6891 (2)	0.86487 (11)	0.0444 (5)	
H7AA	0.2177	0.6638	0.9065	0.053*	
C8A	0.4050 (2)	0.6210 (2)	0.86711 (11)	0.0473 (5)	
C9A	0.3801 (3)	0.4930 (3)	0.88058 (18)	0.0779 (9)	
H9AA	0.3447	0.4553	0.8423	0.117*	
H9AB	0.3268	0.4889	0.9109	0.117*	
H9AC	0.4530	0.4544	0.8971	0.117*	
C10A	0.4563 (3)	0.6847 (3)	0.92732 (16)	0.0710 (9)	
H10A	0.4733	0.7643	0.9176	0.107*	
H10B	0.5280	0.6467	0.9464	0.107*	
H10C	0.3999	0.6835	0.9560	0.107*	
C11A	0.4935 (3)	0.6308 (4)	0.82125 (15)	0.0748 (10)	
H11A	0.5215	0.7107	0.8201	0.090*	
H11B	0.5612	0.5806	0.8348	0.090*	
C1B	0.4490 (2)	0.8726 (2)	0.69862 (10)	0.0405 (5)	
C2B	0.5510 (2)	0.9442 (2)	0.71580 (12)	0.0494 (6)	
H2BA	0.5686	0.9712	0.7570	0.059*	
C3B	0.6240 (2)	0.9745 (3)	0.67371 (13)	0.0532 (6)	
H3BA	0.6914	1.0196	0.6865	0.064*	
C4B	0.5961 (2)	0.9370 (3)	0.61158 (12)	0.0512 (6)	
C5B	0.4966 (2)	0.8706 (2)	0.59204 (11)	0.0466 (5)	
H5BA	0.4778	0.8493	0.5499	0.056*	
C6B	0.4235 (2)	0.8348 (2)	0.63480 (11)	0.0410 (5)	
C7B	0.3283 (2)	0.7547 (2)	0.61084 (10)	0.0418 (5)	
H7BA	0.3106	0.7463	0.5673	0.050*	
C8B	0.1836 (2)	0.6052 (2)	0.61156 (11)	0.0485 (6)	
C9B	0.2573 (4)	0.5016 (3)	0.5970 (2)	0.0877 (12)	
H9BA	0.3069	0.5244	0.5670	0.132*	
H9BB	0.2058	0.4392	0.5798	0.132*	
H9BC	0.3059	0.4754	0.6350	0.132*	
C10B	0.1066 (3)	0.6519 (3)	0.55286 (14)	0.0693 (9)	
H10D	0.0621	0.7179	0.5638	0.104*	
H10E	0.0531	0.5918	0.5348	0.104*	
H10F	0.1560	0.6755	0.5228	0.104*	
C11B	0.1044 (3)	0.5691 (3)	0.65874 (14)	0.0709 (9)	
H11C	0.0464	0.6298	0.6620	0.085*	
H11D	0.0624	0.4977	0.6448	0.085*	
C1S	0.6898 (11)	0.6407 (7)	0.6647 (5)	0.207 (5)	
H1SA	0.7614	0.6721	0.6842	0.248*	
C2S	0.7801 (10)	0.6597 (10)	0.5804 (6)	0.282 (7)	
H2SA	0.8299	0.7096	0.6093	0.423*	
H2SB	0.7546	0.7011	0.5418	0.423*	
H2SC	0.8238	0.5913	0.5720	0.423*	
C3S	0.5866 (8)	0.5788 (7)	0.5676 (5)	0.245 (6)	
H3SA	0.5195	0.5767	0.5895	0.368*	
H3SB	0.6056	0.5008	0.5561	0.368*	
H3SC	0.5679	0.6258	0.5303	0.368*	

### Atomic displacement parameters (Å^2^)

**Table:**

	*U*^11^	*U*^22^	*U*^33^	*U*^12^	*U*^13^	*U*^23^
Ni	0.0460 (2)	0.0483 (3)	0.0325 (2)	−0.00103 (17)	0.01224 (16)	0.00310 (16)
O1A	0.0568 (11)	0.0894 (15)	0.0464 (10)	0.0168 (10)	0.0217 (8)	0.0294 (10)
O2A	0.0693 (12)	0.0940 (15)	0.0410 (9)	0.0308 (11)	0.0169 (9)	0.0006 (10)
O3A	0.0570 (13)	0.139 (2)	0.0816 (17)	0.0211 (14)	0.0310 (12)	0.0058 (16)
O4A	0.095 (2)	0.260 (4)	0.0514 (15)	0.061 (3)	0.0409 (13)	0.037 (2)
O1B	0.0590 (10)	0.0576 (10)	0.0398 (8)	−0.0098 (8)	0.0226 (8)	−0.0105 (8)
O2B	0.1166 (19)	0.0817 (16)	0.0496 (11)	−0.0528 (15)	−0.0091 (12)	0.0236 (11)
O3B	0.0959 (19)	0.233 (4)	0.0830 (18)	−0.101 (3)	0.0347 (15)	−0.025 (2)
O4B	0.0895 (17)	0.144 (3)	0.0539 (13)	−0.0486 (17)	0.0356 (12)	−0.0146 (14)
O1S	0.217 (5)	0.130 (4)	0.268 (7)	0.044 (4)	0.176 (5)	0.052 (4)
N1A	0.0447 (10)	0.0480 (10)	0.0334 (9)	0.0018 (8)	0.0082 (8)	−0.0002 (8)
N2A	0.0567 (14)	0.114 (2)	0.0565 (15)	0.0125 (14)	0.0244 (12)	0.0019 (15)
N1B	0.0418 (9)	0.0437 (10)	0.0341 (9)	−0.0043 (8)	0.0095 (7)	0.0010 (7)
N2B	0.0626 (14)	0.105 (2)	0.0576 (15)	−0.0316 (15)	0.0224 (12)	0.0003 (15)
N1S	0.0749 (19)	0.105 (3)	0.089 (2)	0.0207 (18)	0.0082 (17)	−0.022 (2)
C1A	0.0443 (12)	0.0489 (12)	0.0383 (11)	−0.0007 (10)	0.0118 (9)	0.0044 (10)
C2A	0.0508 (14)	0.0653 (16)	0.0437 (13)	0.0049 (12)	0.0097 (11)	0.0116 (12)
C3A	0.0461 (13)	0.0664 (16)	0.0489 (14)	0.0071 (12)	0.0095 (11)	0.0011 (12)
C4A	0.0492 (13)	0.0673 (16)	0.0432 (13)	0.0007 (12)	0.0153 (11)	−0.0008 (12)
C5A	0.0517 (13)	0.0632 (15)	0.0327 (11)	−0.0004 (12)	0.0128 (10)	0.0020 (11)
C6A	0.0447 (11)	0.0484 (12)	0.0343 (11)	−0.0022 (10)	0.0100 (9)	0.0007 (9)
C7A	0.0499 (12)	0.0564 (14)	0.0275 (10)	−0.0011 (10)	0.0081 (9)	0.0035 (9)
C8A	0.0473 (12)	0.0552 (14)	0.0381 (11)	0.0074 (11)	0.0030 (9)	−0.0033 (10)
C9A	0.087 (2)	0.0625 (19)	0.081 (2)	0.0095 (17)	0.0050 (18)	0.0098 (17)
C10A	0.0604 (17)	0.091 (2)	0.0569 (17)	0.0084 (16)	−0.0045 (14)	−0.0163 (16)
C11A	0.0565 (16)	0.113 (3)	0.0553 (16)	0.0244 (18)	0.0109 (13)	0.0081 (18)
C1B	0.0423 (11)	0.0430 (11)	0.0384 (11)	0.0006 (9)	0.0129 (9)	−0.0035 (9)
C2B	0.0524 (13)	0.0549 (14)	0.0418 (12)	−0.0059 (11)	0.0106 (10)	−0.0100 (11)
C3B	0.0491 (13)	0.0584 (15)	0.0534 (14)	−0.0153 (11)	0.0116 (11)	−0.0055 (12)
C4B	0.0480 (13)	0.0610 (15)	0.0473 (13)	−0.0086 (11)	0.0159 (11)	0.0004 (11)
C5B	0.0475 (12)	0.0569 (14)	0.0371 (11)	−0.0038 (11)	0.0123 (9)	0.0009 (10)
C6B	0.0412 (11)	0.0451 (11)	0.0386 (11)	−0.0018 (9)	0.0116 (9)	0.0001 (9)
C7B	0.0450 (11)	0.0512 (13)	0.0307 (10)	−0.0042 (10)	0.0107 (8)	−0.0003 (9)
C8B	0.0529 (13)	0.0521 (13)	0.0403 (12)	−0.0125 (11)	0.0068 (10)	0.0004 (10)
C9B	0.092 (3)	0.0579 (19)	0.112 (3)	−0.0058 (18)	0.014 (2)	−0.024 (2)
C10B	0.0609 (17)	0.095 (2)	0.0488 (15)	−0.0250 (16)	−0.0020 (13)	0.0124 (16)
C11B	0.0742 (19)	0.089 (2)	0.0488 (15)	−0.0383 (17)	0.0064 (14)	0.0091 (15)
C1S	0.334 (13)	0.135 (6)	0.194 (8)	0.114 (8)	0.173 (9)	0.055 (6)
C2S	0.200 (10)	0.238 (12)	0.45 (2)	0.030 (9)	0.191 (12)	0.004 (12)
C3S	0.198 (8)	0.150 (7)	0.334 (13)	0.077 (6)	−0.126 (9)	−0.084 (8)

### Geometric parameters (Å, °)

**Table:**

Ni—O1A	2.004 (2)	C8A—C9A	1.526 (4)
Ni—O1B	2.0365 (19)	C9A—H9AA	0.9600
Ni—N1A	2.038 (2)	C9A—H9AB	0.9600
Ni—N1B	2.0465 (19)	C9A—H9AC	0.9600
Ni—O2B	2.101 (2)	C10A—H10A	0.9600
Ni—O2A	2.106 (2)	C10A—H10B	0.9600
O1A—C1A	1.273 (3)	C10A—H10C	0.9600
O2A—C11A	1.429 (4)	C11A—H11A	0.9700
O2A—H2A	0.8200	C11A—H11B	0.9700
O3A—N2A	1.222 (4)	C1B—C6B	1.423 (3)
O4A—N2A	1.215 (4)	C1B—C2B	1.423 (3)
O1B—C1B	1.298 (3)	C2B—C3B	1.369 (4)
O2B—C11B	1.420 (4)	C2B—H2BA	0.9300
O2B—H2B	0.8200	C3B—C4B	1.391 (4)
O3B—N2B	1.216 (3)	C3B—H3BA	0.9300
O4B—N2B	1.220 (3)	C4B—C5B	1.375 (4)
O1S—C1S	1.115 (8)	C5B—C6B	1.397 (3)
N1A—C7A	1.278 (3)	C5B—H5BA	0.9300
N1A—C8A	1.494 (3)	C6B—C7B	1.452 (3)
N2A—C4A	1.443 (4)	C7B—H7BA	0.9300
N1B—C7B	1.272 (3)	C8B—C9B	1.514 (5)
N1B—C8B	1.493 (3)	C8B—C10B	1.516 (4)
N2B—C4B	1.446 (3)	C8B—C11B	1.519 (4)
N1S—C1S	1.250 (9)	C9B—H9BA	0.9600
N1S—C2S	1.375 (10)	C9B—H9BB	0.9600
N1S—C3S	1.385 (8)	C9B—H9BC	0.9600
C1A—C6A	1.433 (3)	C10B—H10D	0.9600
C1A—C2A	1.433 (4)	C10B—H10E	0.9600
C2A—C3A	1.352 (4)	C10B—H10F	0.9600
C2A—H2AA	0.9300	C11B—H11C	0.9700
C3A—C4A	1.399 (4)	C11B—H11D	0.9700
C3A—H3AA	0.9300	C1S—H1SA	0.9300
C4A—C5A	1.371 (4)	C2S—H2SA	0.9600
C5A—C6A	1.396 (3)	C2S—H2SB	0.9600
C5A—H5AA	0.9300	C2S—H2SC	0.9600
C6A—C7A	1.448 (3)	C3S—H3SA	0.9600
C7A—H7AA	0.9300	C3S—H3SB	0.9600
C8A—C10A	1.519 (4)	C3S—H3SC	0.9600
C8A—C11A	1.523 (4)		
			
O1A—Ni—O1B	92.52 (9)	C8A—C10A—H10B	109.5
O1A—Ni—N1A	90.16 (8)	H10A—C10A—H10B	109.5
O1B—Ni—N1A	98.60 (8)	C8A—C10A—H10C	109.5
O1A—Ni—N1B	93.48 (8)	H10A—C10A—H10C	109.5
O1B—Ni—N1B	89.22 (7)	H10B—C10A—H10C	109.5
N1A—Ni—N1B	171.23 (8)	O2A—C11A—C8A	108.8 (3)
O1A—Ni—O2B	87.84 (11)	O2A—C11A—H11A	109.9
O1B—Ni—O2B	167.86 (8)	C8A—C11A—H11A	109.9
N1A—Ni—O2B	93.54 (8)	O2A—C11A—H11B	109.9
N1B—Ni—O2B	78.64 (8)	C8A—C11A—H11B	109.9
O1A—Ni—O2A	168.90 (8)	H11A—C11A—H11B	108.3
O1B—Ni—O2A	90.31 (9)	O1B—C1B—C6B	123.3 (2)
N1A—Ni—O2A	78.79 (8)	O1B—C1B—C2B	119.5 (2)
N1B—Ni—O2A	97.30 (8)	C6B—C1B—C2B	117.3 (2)
O2B—Ni—O2A	91.64 (11)	C3B—C2B—C1B	122.3 (2)
C1A—O1A—Ni	126.42 (16)	C3B—C2B—H2BA	118.9
C11A—O2A—Ni	106.24 (17)	C1B—C2B—H2BA	118.9
C11A—O2A—H2A	109.5	C2B—C3B—C4B	119.0 (2)
Ni—O2A—H2A	120.9	C2B—C3B—H3BA	120.5
C1B—O1B—Ni	125.03 (15)	C4B—C3B—H3BA	120.5
C11B—O2B—Ni	107.94 (18)	C5B—C4B—C3B	121.1 (2)
C11B—O2B—H2B	109.5	C5B—C4B—N2B	118.7 (2)
Ni—O2B—H2B	141.8	C3B—C4B—N2B	120.1 (2)
C7A—N1A—C8A	119.1 (2)	C4B—C5B—C6B	120.8 (2)
C7A—N1A—Ni	124.07 (17)	C4B—C5B—H5BA	119.6
C8A—N1A—Ni	115.76 (15)	C6B—C5B—H5BA	119.6
O4A—N2A—O3A	122.1 (3)	C5B—C6B—C1B	119.5 (2)
O4A—N2A—C4A	118.3 (3)	C5B—C6B—C7B	116.3 (2)
O3A—N2A—C4A	119.5 (3)	C1B—C6B—C7B	124.1 (2)
C7B—N1B—C8B	118.5 (2)	N1B—C7B—C6B	126.1 (2)
C7B—N1B—Ni	125.61 (17)	N1B—C7B—H7BA	116.9
C8B—N1B—Ni	115.59 (14)	C6B—C7B—H7BA	116.9
O3B—N2B—O4B	122.2 (3)	N1B—C8B—C9B	107.4 (2)
O3B—N2B—C4B	118.1 (3)	N1B—C8B—C10B	112.8 (2)
O4B—N2B—C4B	119.6 (2)	C9B—C8B—C10B	111.8 (3)
C1S—N1S—C2S	116.5 (9)	N1B—C8B—C11B	106.3 (2)
C1S—N1S—C3S	125.0 (9)	C9B—C8B—C11B	109.5 (3)
C2S—N1S—C3S	118.5 (8)	C10B—C8B—C11B	108.9 (2)
O1A—C1A—C6A	124.0 (2)	C8B—C9B—H9BA	109.5
O1A—C1A—C2A	119.1 (2)	C8B—C9B—H9BB	109.5
C6A—C1A—C2A	116.8 (2)	H9BA—C9B—H9BB	109.5
C3A—C2A—C1A	122.4 (2)	C8B—C9B—H9BC	109.5
C3A—C2A—H2AA	118.8	H9BA—C9B—H9BC	109.5
C1A—C2A—H2AA	118.8	H9BB—C9B—H9BC	109.5
C2A—C3A—C4A	119.2 (2)	C8B—C10B—H10D	109.5
C2A—C3A—H3AA	120.4	C8B—C10B—H10E	109.5
C4A—C3A—H3AA	120.4	H10D—C10B—H10E	109.5
C5A—C4A—C3A	121.1 (2)	C8B—C10B—H10F	109.5
C5A—C4A—N2A	120.1 (2)	H10D—C10B—H10F	109.5
C3A—C4A—N2A	118.8 (3)	H10E—C10B—H10F	109.5
C4A—C5A—C6A	120.8 (2)	O2B—C11B—C8B	109.1 (2)
C4A—C5A—H5AA	119.6	O2B—C11B—H11C	109.9
C6A—C5A—H5AA	119.6	C8B—C11B—H11C	109.9
C5A—C6A—C1A	119.5 (2)	O2B—C11B—H11D	109.9
C5A—C6A—C7A	116.5 (2)	C8B—C11B—H11D	109.9
C1A—C6A—C7A	124.1 (2)	H11C—C11B—H11D	108.3
N1A—C7A—C6A	126.2 (2)	O1S—C1S—N1S	131.3 (14)
N1A—C7A—H7AA	116.9	O1S—C1S—H1SA	114.3
C6A—C7A—H7AA	116.9	N1S—C1S—H1SA	114.3
N1A—C8A—C10A	112.0 (2)	N1S—C2S—H2SA	109.5
N1A—C8A—C11A	105.7 (2)	N1S—C2S—H2SB	109.5
C10A—C8A—C11A	108.1 (3)	H2SA—C2S—H2SB	109.5
N1A—C8A—C9A	109.2 (2)	N1S—C2S—H2SC	109.5
C10A—C8A—C9A	110.9 (3)	H2SA—C2S—H2SC	109.5
C11A—C8A—C9A	110.9 (3)	H2SB—C2S—H2SC	109.5
C8A—C9A—H9AA	109.5	N1S—C3S—H3SA	109.5
C8A—C9A—H9AB	109.5	N1S—C3S—H3SB	109.5
H9AA—C9A—H9AB	109.5	H3SA—C3S—H3SB	109.5
C8A—C9A—H9AC	109.5	N1S—C3S—H3SC	109.5
H9AA—C9A—H9AC	109.5	H3SA—C3S—H3SC	109.5
H9AB—C9A—H9AC	109.5	H3SB—C3S—H3SC	109.5
C8A—C10A—H10A	109.5		
			
O1B—Ni—O1A—C1A	−123.2 (2)	C2A—C1A—C6A—C5A	−2.6 (4)
N1A—Ni—O1A—C1A	−24.5 (2)	O1A—C1A—C6A—C7A	−1.7 (4)
N1B—Ni—O1A—C1A	147.5 (2)	C2A—C1A—C6A—C7A	178.7 (2)
O2B—Ni—O1A—C1A	69.0 (2)	C8A—N1A—C7A—C6A	−178.2 (2)
O2A—Ni—O1A—C1A	−18.5 (7)	Ni—N1A—C7A—C6A	−10.8 (4)
O1A—Ni—O2A—C11A	−40.2 (6)	C5A—C6A—C7A—N1A	178.5 (3)
O1B—Ni—O2A—C11A	64.6 (2)	C1A—C6A—C7A—N1A	−2.8 (4)
N1A—Ni—O2A—C11A	−34.1 (2)	C7A—N1A—C8A—C10A	−57.3 (3)
N1B—Ni—O2A—C11A	153.9 (2)	Ni—N1A—C8A—C10A	134.3 (2)
O2B—Ni—O2A—C11A	−127.3 (2)	C7A—N1A—C8A—C11A	−174.7 (3)
O1A—Ni—O1B—C1B	−121.5 (2)	Ni—N1A—C8A—C11A	16.9 (3)
N1A—Ni—O1B—C1B	148.0 (2)	C7A—N1A—C8A—C9A	66.0 (3)
N1B—Ni—O1B—C1B	−28.0 (2)	Ni—N1A—C8A—C9A	−102.5 (2)
O2B—Ni—O1B—C1B	−30.0 (6)	Ni—O2A—C11A—C8A	53.9 (3)
O2A—Ni—O1B—C1B	69.3 (2)	N1A—C8A—C11A—O2A	−46.3 (3)
O1A—Ni—O2B—C11B	62.1 (2)	C10A—C8A—C11A—O2A	−166.4 (3)
O1B—Ni—O2B—C11B	−29.9 (6)	C9A—C8A—C11A—O2A	71.9 (3)
N1A—Ni—O2B—C11B	152.1 (2)	Ni—O1B—C1B—C6B	28.5 (3)
N1B—Ni—O2B—C11B	−31.9 (2)	Ni—O1B—C1B—C2B	−152.63 (19)
O2A—Ni—O2B—C11B	−129.0 (2)	O1B—C1B—C2B—C3B	179.8 (3)
O1A—Ni—N1A—C7A	19.7 (2)	C6B—C1B—C2B—C3B	−1.3 (4)
O1B—Ni—N1A—C7A	112.2 (2)	C1B—C2B—C3B—C4B	1.8 (4)
O2B—Ni—N1A—C7A	−68.2 (2)	C2B—C3B—C4B—C5B	0.2 (4)
O2A—Ni—N1A—C7A	−159.2 (2)	C2B—C3B—C4B—N2B	−177.1 (3)
O1A—Ni—N1A—C8A	−172.58 (18)	O3B—N2B—C4B—C5B	−173.0 (4)
O1B—Ni—N1A—C8A	−80.00 (18)	O4B—N2B—C4B—C5B	3.4 (5)
O2B—Ni—N1A—C8A	99.57 (18)	O3B—N2B—C4B—C3B	4.3 (5)
O2A—Ni—N1A—C8A	8.60 (17)	O4B—N2B—C4B—C3B	−179.3 (3)
O1A—Ni—N1B—C7B	105.4 (2)	C3B—C4B—C5B—C6B	−2.7 (4)
O1B—Ni—N1B—C7B	12.9 (2)	N2B—C4B—C5B—C6B	174.6 (3)
O2B—Ni—N1B—C7B	−167.5 (2)	C4B—C5B—C6B—C1B	3.2 (4)
O2A—Ni—N1B—C7B	−77.3 (2)	C4B—C5B—C6B—C7B	−173.6 (2)
O1A—Ni—N1B—C8B	−80.53 (18)	O1B—C1B—C6B—C5B	177.6 (2)
O1B—Ni—N1B—C8B	−173.01 (17)	C2B—C1B—C6B—C5B	−1.3 (3)
O2B—Ni—N1B—C8B	6.57 (18)	O1B—C1B—C6B—C7B	−5.9 (4)
O2A—Ni—N1B—C8B	96.78 (18)	C2B—C1B—C6B—C7B	175.3 (2)
Ni—O1A—C1A—C6A	19.7 (4)	C8B—N1B—C7B—C6B	−171.9 (2)
Ni—O1A—C1A—C2A	−160.7 (2)	Ni—N1B—C7B—C6B	2.0 (4)
O1A—C1A—C2A—C3A	−177.8 (3)	C5B—C6B—C7B—N1B	165.8 (2)
C6A—C1A—C2A—C3A	1.8 (4)	C1B—C6B—C7B—N1B	−10.8 (4)
C1A—C2A—C3A—C4A	0.4 (5)	C7B—N1B—C8B—C9B	75.3 (3)
C2A—C3A—C4A—C5A	−1.9 (5)	Ni—N1B—C8B—C9B	−99.2 (3)
C2A—C3A—C4A—N2A	175.4 (3)	C7B—N1B—C8B—C10B	−48.3 (3)
O4A—N2A—C4A—C5A	−0.8 (5)	Ni—N1B—C8B—C10B	137.2 (2)
O3A—N2A—C4A—C5A	177.3 (3)	C7B—N1B—C8B—C11B	−167.5 (2)
O4A—N2A—C4A—C3A	−178.1 (4)	Ni—N1B—C8B—C11B	18.0 (3)
O3A—N2A—C4A—C3A	0.1 (5)	Ni—O2B—C11B—C8B	51.5 (3)
C3A—C4A—C5A—C6A	1.0 (4)	N1B—C8B—C11B—O2B	−45.2 (4)
N2A—C4A—C5A—C6A	−176.2 (3)	C9B—C8B—C11B—O2B	70.6 (3)
C4A—C5A—C6A—C1A	1.2 (4)	C10B—C8B—C11B—O2B	−166.9 (3)
C4A—C5A—C6A—C7A	180.0 (3)	C2S—N1S—C1S—O1S	179.8 (8)
O1A—C1A—C6A—C5A	177.0 (3)	C3S—N1S—C1S—O1S	−1.7 (12)

### Hydrogen-bond geometry (Å, °)

**Table:**

*D*—H···*A*	*D*—H	H···*A*	*D*···*A*	*D*—H···*A*
O2A—H2A···O1S	0.82	1.94	2.734 (5)	163.
O2B—H2B···O1B^i^	0.82	1.81	2.619 (3)	166.
C9A—H9AA···O1A^i^	0.96	2.59	3.422 (4)	146.
C11A—H11B···O3B^ii^	0.97	2.50	3.394 (4)	153.
C5B—H5BA···O4A^iii^	0.93	2.57	3.418 (4)	151.

Symmetry codes: (i) −*x*+1/2, *y*−1/2, −*z*+3/2; (ii) −*x*+3/2, *y*−1/2, −*z*+3/2; (iii) *x*+1/2, −*y*+3/2, *z*−1/2.
